# Telomeres, oxidative stress and inflammatory factors: partners in cellular senescence?

**DOI:** 10.1186/2046-2395-3-1

**Published:** 2014-01-16

**Authors:** Clara Correia-Melo, Graeme Hewitt, João F Passos

**Affiliations:** 1 Ageing Research Laboratories, Centre for Integrated Systems Biology of Ageing and Nutrition, Institute for Ageing and Health, Campus for Ageing and Vitality, Newcastle University, Newcastle upon Tyne NE4 5PL, UK; 2 Graduate Programme in Areas of Basic and Applied Biology (GABBA), Institute of Biomedical Sciences Abel Salazar, University of Porto, Porto 4050-313, Portugal

**Keywords:** DNA damage response, Telomeres, Senescence, Ageing, Oxidative stress, Inflammation

## Abstract

Senescence, the state of irreversible cell-cycle arrest, plays paradoxical albeit important roles *in vivo*: it protects organisms against cancer but also contributes to age-related loss of tissue function. The DNA damage response (DDR) has a central role in cellular senescence. Not only does it contribute to the irreversible loss of replicative capacity but also to the production and secretion of reactive oxygen species (ROS), and bioactive peptides collectively known as the senescence-associated secretory phenotype (SASP). Both ROS and the SASP have been shown to impact on senescence in an autocrine as well as paracrine fashion; however, the underlying mechanisms are not well understood. In this review we describe our current understanding of cellular senescence, examine in detail the intricate pathways linking the DDR, ROS and SASP, and evaluate their impact on the stability of the senescent phenotype.

## Review

### Introduction

Cellular senescence, the state of irreversible cell cycle arrest described by Hayflick and Moorhead [1] over 50 years ago, remains an intriguing biological process. Senescence is characterised by dramatic changes in cell morphology, including increased cellular volume and flattening of the cytoplasm [2]. The senescent phenotype also results in changes in nuclear structure, gene expression, protein processing and metabolism, and resistance to apoptosis [3-6].

Whether senescence exists to any significant extent *in vivo* has been the subject of a longstanding debate [7]. In the past decade, remarkable advances have been made demonstrating that senescence plays an important role *in vivo*. Several studies suggest that senescence can act as a tumour suppressor mechanism [8,9]. On the other hand, numerous lines of evidence indicate that senescence can, in the long run, have adverse effects, by impairing organ regeneration and releasing a host of bioactive molecules, including reactive oxygen species (ROS) and a wide variety of pro-inflammatory cytokines, chemokines and growth factors (collectively referred to as the senescence-associated secretory phenotype (SASP)).

Senescent cells containing telomere-induced foci have been shown to increase with age in the skin of baboons, which have similar telomere length to humans and absence of telomerase activity [10]. In mice, cells bearing senescent markers have been reported to increase with age in a variety of tissues [11-13], including post-mitotic neurons [14]. Moreover, senescent cells have been associated with several age-related diseases, such as diabetes [15] and atherosclerosis [16]. While noteworthy, these data do not provide causality. A major challenge in the field has been to determine if and how senescent cells contribute to age-related tissue dysfunction, or if they merely correlate with it.

Mounting evidence indicates that activation of pathways involved in cellular senescence impacts on mammalian lifespan [17-19]. Recently, the van Deursen group has shown that inducible elimination of p16Ink4a-positive senescent cells from the eye, adipose and skeletal tissues in the BubR1 progeroid mouse model delayed acquisition of age-related pathologies in these tissues. They showed that elimination of p16Ink4a-positive cells also attenuated the progression of already established age-related disorders, suggesting that cellular senescence may have a causal role in age-related tissue impairment [20].

Though several mechanisms responsible for the activation of senescence have been identified, it is still unclear how a cell “commits” to becoming irreversibly arrested. Recent studies have revealed that the SASP, as well as mitochondrial/metabolic alterations, may contribute to the reinforcement of the growth arrest via a series of positive feedback loops involving a persistent activation of the DNA damage response (DDR) [21-23].

The aim of this review is to describe the current understanding of cellular senescence, providing special focus on the intricate pathways that link the nucleus, mitochondria and secreted proteins, and contribute to the stability of the senescent phenotype.

### Telomeres and the stabilisation of cellular senescence

Telomeres are regions of DNA and associated proteins present at the end of linear chromosomes; in vertebrates they are tandem repeats of the sequence TTAGGG [24].

Telomeres are bound by a group of telomere-associated proteins known as the “shelterin” complex [25]. These proteins are thought to arrange telomeric DNA into a loop structure known as the T-loop [26]. This structure was first visualised in purified telomere restriction fragments using electron microscopy, and it is proposed to prevent the activation of a DDR by hiding the exposed DNA ends. The shelterin complex is comprised of six proteins: TRF1, TRF2 and POT1, which recognise the telomeric repeat sequence, and additional proteins TIN2, TPP1 and Rap1 [25].

Telomere shortening is probably the best studied mechanism driving cellular senescence. It mainly occurs during cell division due to the inability of the DNA replication machinery, specifically DNA polymerase, to synthesise in a 3′-5′ direction leading to the incomplete replication of the lagging strand. It has been shown that telomere shortening contributes causally to cellular senescence, since overexpression of telomerase, an enzyme able to maintain telomere length, resulted in cell immortalisation [27]. Mouse models, where telomere function has been compromised, strongly support a role for senescence (and telomeres) in the ageing process. Telomerase knock-out (mTERC-/-) mice which carry a homozygous deletion of the RNA component of telomerase [28] show a progressive generation-dependent telomere shortening, which results in both cell-cycle arrest and apoptosis [29]. Telomere dysfunction in mTERC-/- mice has been shown to limit stem cell function, regeneration, organ homeostasis and lifespan [30].

It is believed that the progressive loss of telomere repeats destabilises T-loops [26] and, as a consequence, increases the probability of telomere uncapping (that is, loss of “shelterin”). Uncapping of telomeres, whether by inhibition of TRF2 or telomere shortening, has been shown to activate the DDR in a manner similar to DNA double strand breaks (DSBs) [31,32]. The DDR can elicit a transient cell-cycle arrest, allowing sufficient time for the cellular repair machinery to act and repair the DNA damage [33]. However, if the damage is irreparable, the arrest can become permanent. This response is initiated by the phosphatidylinositol 3-kinase-like protein kinases ATM and ATR, which phosphorylate proteins such as H2A.X and NBS1, and downstream kinases CHK1 and CHK2, which ultimately activate p53 and p21 proteins [34]. Several groups have reported that senescence is characterised by a persistent activation of the DDR, which is necessary for both the development and stability of the phenotype [21,35].

One important question is: what contributes to a persistent DDR during cellular senescence? Recent work has highlighted the importance of telomeres in the maintenance of senescence. It has been demonstrated that DNA damage at telomeres can occur as a consequence of genotoxic and oxidative stress, and that this damage is mostly irreparable [13,36]. In order to establish whether a telomeric location is necessary for foci to persist, using live-cell imaging, our group has tracked the lifespan of DNA damage foci using a AcGFP-53BP1c fusion protein in combination with a fluorescently labelled PNA probe that specifically tags telomere repeats. Using this method it was found that the majority of long-lived foci in stress-induced senescent cells co-localise with telomeres [13], which suggests that they are major contributors to a persistent DDR.

These findings raise questions regarding how the cellular repair machinery distinguishes telomeres and DSBs. Non-homologous end joining (NHEJ) is strongly inhibited in telomeric regions, perhaps as a mechanism to prevent end-to-end fusions [37]. NHEJ is the major pathway for the repair of DSBs. Moreover, displacement of TRF2 from telomeres by overexpression of TRF2^ΔBΔM^, or conditional deletion of TRF2, has been shown to result in telomere fusions [37-39]. It has also been demonstrated *in vitro* that TRF2 and its binding partner RAP1 are required to prevent NHEJ-dependent telomeric DNA fusions by inhibiting DNA-PK and ligase IV mediated end-joining [40]. Consistent with these data, Fumagalli and colleagues have shown in budding yeast that induction of a DNA DSB adjacent to a telomeric sequence impairs the recruitment of ligase IV to the site of damage [36]. This suggests that damage at telomeres, occurring in the presence of sufficient shelterin components including TRF2, may elicit a persistent DDR due to inhibition of repair. In accordance with this hypothesis, it has been shown recently that during replicative senescence of human fibroblasts, telomeres positive for DDR retain both TRF2 and RAP1 and are not associated with end-to-end fusions [41].

Recent studies have shown that the role of telomeres in senescence may extend beyond attrition due to replication. A recent study has shown that oncogenic signals cause replication fork stalling, resulting in telomeric DNA damage accumulation, activation of a DDR and consequently senescence [42]. However, it has been reported that in both replicative and stress-induced senescent cells, 50% of DNA damage foci can be found in non-telomeric regions of the genome and are short-lived. Live-cell imaging studies have shown that these short-lived foci are maintained in relatively constant numbers per cell and that new foci are regularly being created during senescence [13,21]. Moreover, data indicate that these foci are mainly the result of ROS production during senescence and contribute to some degree to the stability and development of the phenotype. Consistently, following the activation of a DDR, inhibition of ROS production results in a small fraction of cells being able to resume proliferation [21].

**Figure 1:**
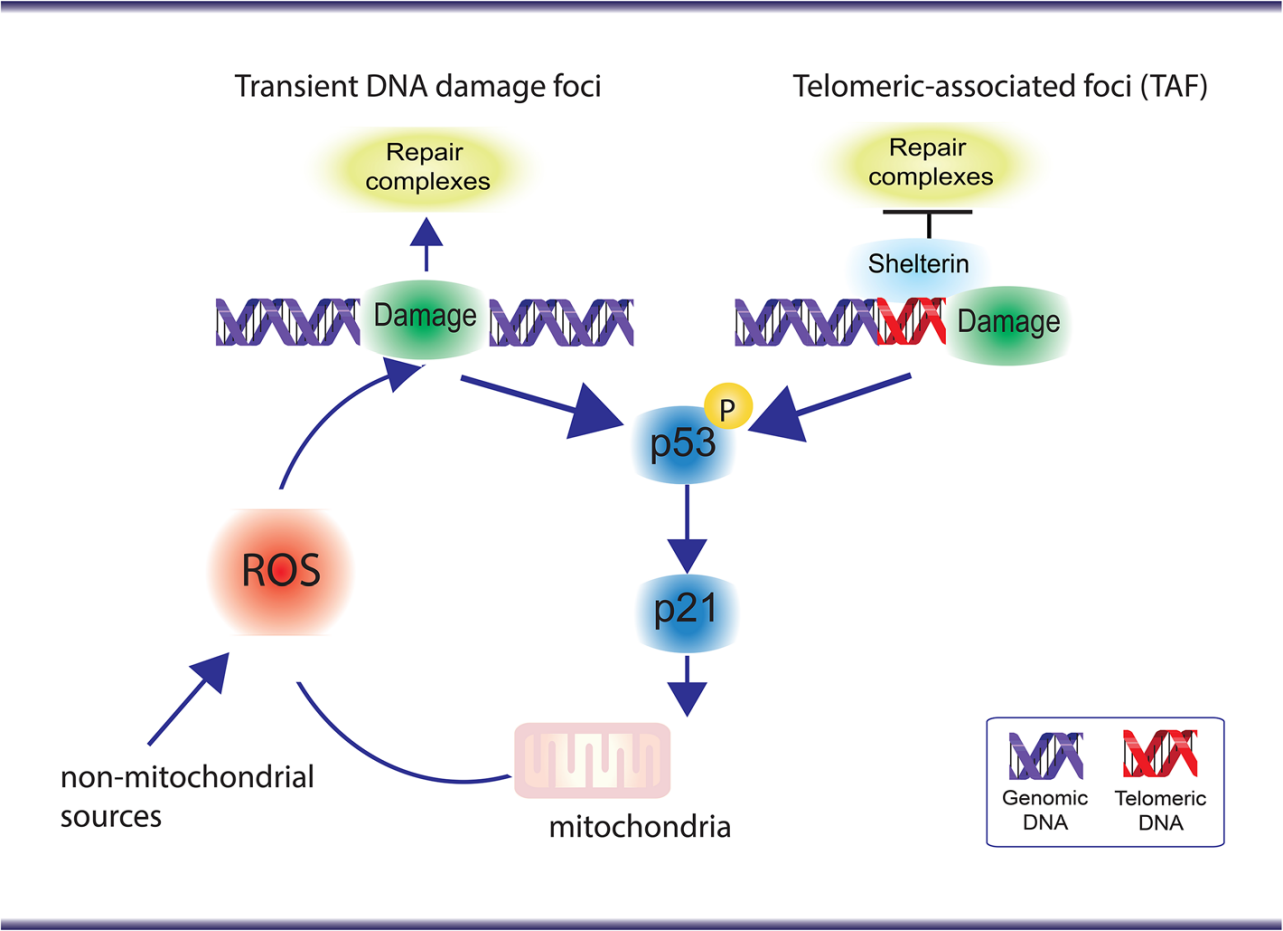
**Both telomeric and non-telomeric DNA damage contribute to the stabilisation of cellular senescence.** DNA damage at telomeres is distinct from that throughout the genome; it is irreparable due to the repression of DNA repair pathways by telomere bound proteins, known as the “shelterin” complex. This contributes to a permanent DNA damage response (DDR). However, continuous generation of short-lived DDR foci by elevated reactive oxygen species (ROS) may equally contribute to the maintenance of the phenotype, as long as a dynamic equilibrium between damage induction and repair can be maintained.

Therefore, it is highly likely that both telomeric and non-telomeric regions are contributors to the senescent phenotype (Figure 1); however, their relative contribution towards senescence signalling is experimentally very difficult to dissect.

Importantly, mechanisms other than the DDR have been shown to impact on the stability of the senescent phenotype. In several types of cells, senescence is accompanied by drastic changes in chromatin organisation, such as formation of senescence-associated heterochromatic foci, which are dependent on the p16/Rb pathway [6]. Senescence-associated heterochromatic foci have been shown to accumulate on the promoters of cell-cycle genes during senescence, and their occurrence has been shown to correlate with the irreversibility of the senescent phenotype [6,43].

### Involvement of reactive oxygen species in the stabilisation of cellular senescence

**Figure 2:**
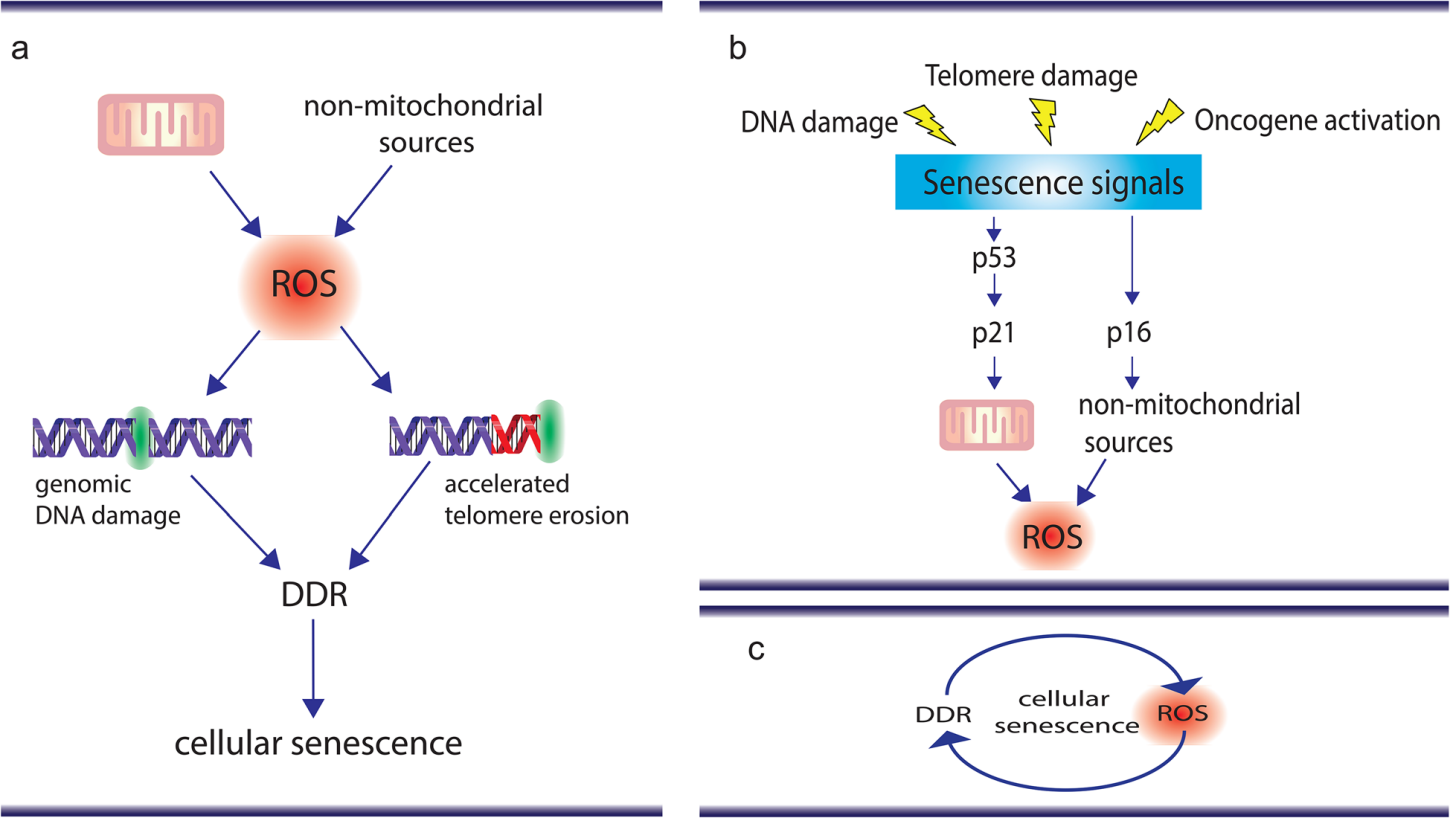
**Two different models by which reactive oxygen species can impact on cellular senescence. (a)** Reactive oxygen species (ROS) produced via mitochondrial and non-mitochondrial sources can induce genomic DNA damage and accelerate telomere erosion/damage, both of which contribute to activation of a DNA damage response (DDR). **(b)** ROS can act as signalling molecules in senescence: activation of “senescence signals” has been shown to result in increased ROS generation (mitochondrial and non-mitochondrial). ROS has been shown to impact on a variety of pathways which may help stabilise the senescence growth arrest. **(c)** Simplified feedback loop model involving ROS and DNA damage. Telomere uncapping or general DNA damage triggers a DDR which culminates through yet unidentified processes to ROS generation. ROS generation leads to additional DNA damage to the genome, stabilising the DDR and leading to a stable senescence arrest.

ROS are likely to be involved in both the induction and stabilisation of cellular senescence: several studies have shown that ROS can accelerate telomere shortening [44], and can damage DNA directly and thus induce a DDR and senescence [45-47] (Figure 2a). ROS have been implicated in organismal ageing, with countless reports of associations between oxidative damage and the ageing process [48-50]; however, genetically manipulated animal models where mitochondrial function and oxidative stress were targeted have generated conflicting results [51].

Several studies have shown that cellular senescence is characterised by mitochondrial dysfunction contributing to metabolic inefficiency and elevated ROS [52-56]. Elevated ROS levels have been associated with replicative, stress- and oncogene-induced senescence [8,45,55,57].

Evidence indicates that activation of major downstream effectors of the DDR in senescence result in elevated ROS. Activation of a DDR by genotoxic stress or telomere uncapping [21], over-expression of activated RAS [58], BRAF^V600E^[59], p53 [60], p21 [61] and p16 [62] all resulted in elevated ROS generation. In most of the above reported cases treatment with antioxidants, such as N-acetyl cysteine, were able to prevent the cell-cycle arrest supporting a causal role for ROS in the process (Figure 2b).

These data indicate that elevated ROS are a consequence of the activation of the senescence programme and has led to the suggestion that ROS may act as signalling molecules during cellular senescence [63]. However, mechanistically it is still unclear how these pathways contribute to mitochondrial dysfunction and ROS generation. Takahashi and colleagues, using human fibroblasts expressing a temperature-sensitive simian virus 40 large T antigen, connected p16 with ROS production via protein kinase Cδ signalling [62]. Protein kinase Cδ has been shown to activate a non-mitochondrial source of ROS, generated by NADPH-oxidase through phosphorylation of p47^phox^, an essential component of NADPH oxidase [64]. Consistent with this study, NADPH oxidases have been shown to limit the replicative lifespan of human endothelial cells in culture via ROS generation [65].

Oncogene-induced senescence has been associated with mitochondrial dysfunction and ROS production, which is dependent on intact p53 and Rb tumour suppression pathways. Mitochondrial dysfunction resulted in the loss of ATP and activation of AMPK; in addition, mitochondrial-derived ROS were shown to contribute to the oxidation of DNA [66]. In a recent study, it was shown that BRAF^V600E^-induced senescence was accompanied by the activation of pyruvate dehydrogenase, which resulted in the enhanced use of pyruvate by the tricarboxylic acid cycle followed by increased respiration and ROS generation [59].

The role of p53 and p21 in ROS generation during senescence is still not well understood. An association between p53 and transcriptional activation of genes involved in mitochondrial apoptosis has been demonstrated [67], as well as a stress-induced translocation of p53 to mitochondria resulting in increased outer membrane permeabilisation [68]; however, a direct role of mitochondrial p53 in cellular senescence has not yet been demonstrated. In contrast, transcriptional regulation of mitochondrial genes by p53 has been reported to impact on mitochondrial function and contribute to ageing. p53 knock-out mice exhibited reduced expression of the *Sco2* gene, which is required for the assembly of the mitochondrial DNA-encoded COX II subunit [69]. In late generation telomerase knock-out mice that have critically short telomeres, activation of p53 has been shown to repress the promoters of *PGC*-*1α* and *PGC*-*1β* genes, master regulators of mitochondrial biogenesis and function, thereby contributing to decreased mitochondrial function [70].

Knockdown of both p53 and p21 by RNA-mediated interference has been shown to reduce ROS generation in both telomere-dependent and -independent senescence [21]. Our group has found that ROS levels increase in senescent cells as a result of signalling through p21, and feed back into DNA damage induction and the DDR, generating a stable, self-sustaining feedback loop (Figure 2c). This feedback loop persists even in irreversibly deep senescence. Moreover, p21 appears to be the critical mediator between the DDR and MAPK and transforming growth factor (TGF)-*β* stress-induced signalling cascades, which have been shown to contribute to ROS generation [21,71,72]. Consistently, a p21 knock-out rescued at least some accelerated ageing phenotypes in telomerase (mTERC) knock-out mice [17], as well as markers of oxidative stress and DNA damage foci [21]. ROS has also been shown to impact on the DDR and ultimately senescence in a non-cell-autonomous fashion. A recent study has shown that senescent cells can induce a DDR in neighbouring cells via a gap junction-mediated cell-cell contact and processes involving ROS [73].

### Synergistic interactions between the senescence-associated secretory phenotype and reactive oxygen species during senescence

During senescence, another major contributor to the stabilisation of the growth arrest is mediated by autocrine signalling involving the secretion of bioactive, frequently pro-inflammatory peptides, known as the SASP [74] or senescence-messaging secretome [75]. The SASP includes several families of soluble and insoluble factors. The soluble factors include signalling molecules such as growth factors, inflammatory and immune-modulatory cytokines and chemokines, whereas the insoluble factors mainly comprise extracellular matrix components [76]. It has long been recognised that the primary function of secreted factors is to allow inter- and intra-cellular communication. However, the SASP has been found to play a series of somewhat contradictory roles, with important consequences for ageing and cancer. First, it can contribute to the surveillance and elimination of senescent cells by the immune system [77,78]. Second, it can be pro-tumorigenic [74,79,80]; both cell culture experiments and studies involving the co-transplantation of senescent and cancer cells into recipient mice have shown that senescent fibroblasts can stimulate the hyperproliferation of cancer cells, neoplastic progression and tissue damage. Third, it can contribute to the reinforcement of oncogene- or stress-induced senescence in a cell-autonomous fashion [22,23]. Fourth, it can induce senescence in neighbouring cells via a bystander effect both *in vitro* and *in vivo*[81].

**Figure 3:**
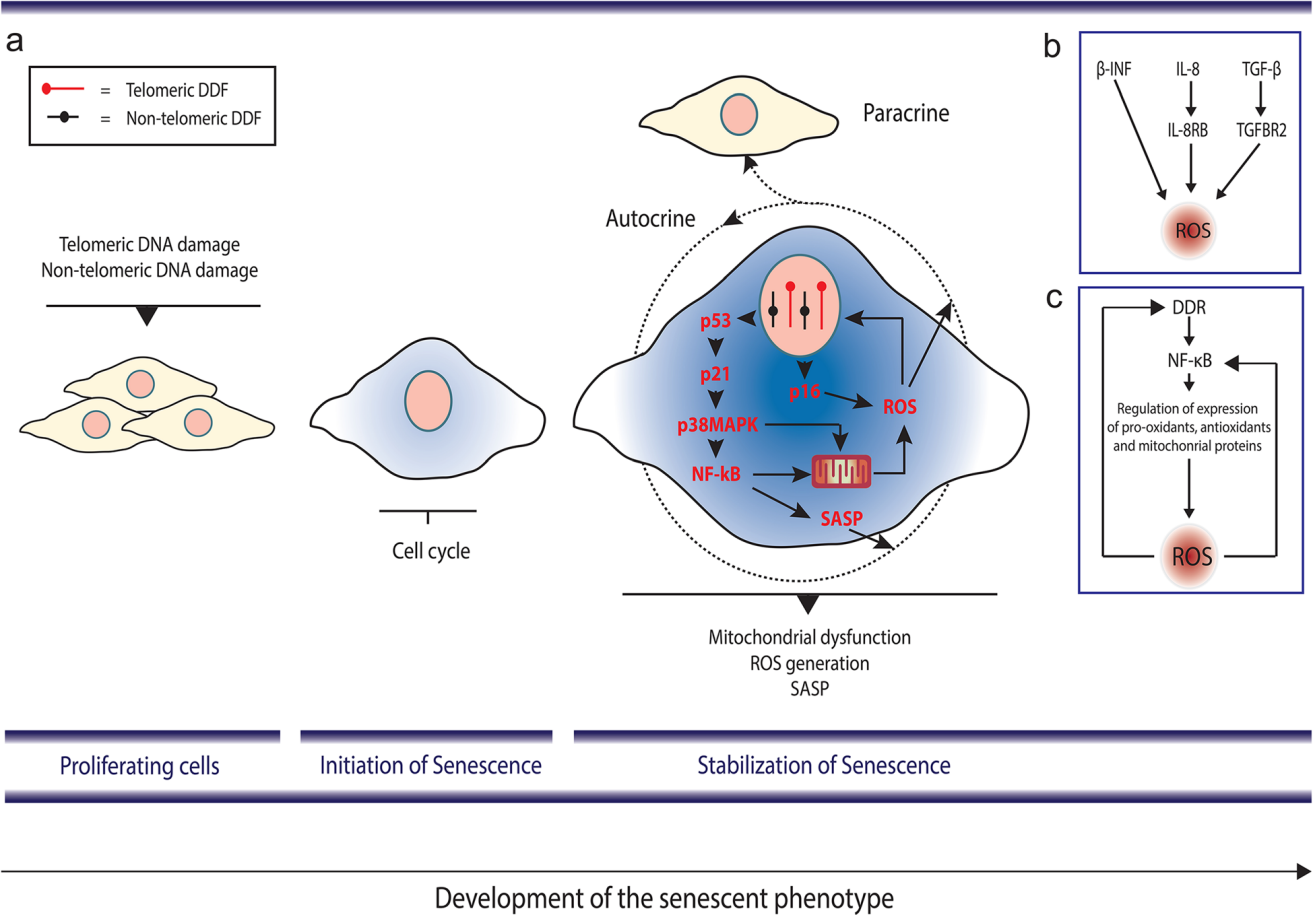
**Senescence is a multi-layered process involving interactions between the DNA damage response, reactive oxygen species and senescence-associated secretory phenotype. (a)** Initially, stressors such as telomeric and non-telomeric DNA damage can lead to activation of a DNA damage response (DDR) and cell cycle arrest. Following activation of the DDR, p53, p21 and p38MAPK pathways have been shown to enhance nuclear factor (NF)-κB transcriptional activity. NF-κB activation is both responsible for the senescence-associated secretory phenotype (SASP) and can induce (and be activated) by reactive oxygen species (ROS). p16 has been shown to induce ROS generation via NADPH oxidases [62]; however, it has been shown to be unrelated to the SASP [88]. Secretion of bioactive molecules such as ROS and SASP factors contribute not only to reinforce senescence in an autocrine fashion, but also to induce senescence in neighbouring cells. **(b)** Components of the SASP (such as IL-8, β-IFN and transforming growth factor (TGF)-β) have been shown to reinforce the senescence arrest via ROS through yet unidentified mechanisms [21,22,89]. **(c)** NF-κB transcriptional activity has been shown to be dependent on the DDR and ROS. However, NF-κB activation has been shown to increase ROS generation (via regulating expression of mitochondrial genes or antioxidant, pro-oxidant genes) [96,97]. DDF - DNA Damage Foci.

Mechanistically, it is still not entirely understood how the SASP contributes to the reinforcement of senescence; however, several lines of evidence suggest the existence of synergistic interactions between the DDR, ROS and inflammatory signals (Figure 3a). Kinetic analysis has shown that ROS levels increase 2 to 3 days following activation of a DDR [21], while the SASP occurs 7 to 10 days later [76]. Induction of both ROS and the SASP in X-ray irradiation-induced senescence has been shown to be dependent on activation of the DDR [21,35].

The nuclear factor (NF)-κB family of transcriptional factors regulate expression of numerous genes involved in a variety of cellular processes including stress response and inflammation [82]. Importantly, activation of NF-κB has been considered critical in chronic inflammatory diseases by increasing the expression of the genes for many cytokines, enzymes, and adhesion molecules [83]. Increased NF-κB activity has been shown to play an important role in senescence [84] and the SASP [85].

Recent investigations using progeroid mouse models (models of premature ageing) driven by DNA damage have reported that these mice have increased activation of NF-κB driven chronic inflammation and senescence [86,87]. Interestingly, in a murine model of XFE (xeroderma pigmentosum F–excision repair) progeroid syndrome, *Ercc1*^–/*Δ*^ mice, inhibition of NF-κB signalling not only reduced the onset of several age-related pathologies, but also both DNA and protein oxidation [87], suggesting a potential link between inflammation and ROS pathways.

Another link between ROS and the SASP during senescence involves the p38 mitogen-activated protein kinase (p38MAPK). p38MAPK has been shown to regulate the SASP in senescence mainly through NF-κB transcriptional activity [85]. Similarly, the p38MAPK pathway has been shown to be important for ROS generation in both stress-induced and replicative senescence and for the stability of the DDR [21]. p16, an important tumour suppressor gene which can be induced by stresses other than DNA damage, has been linked to increased ROS production [62]; however, less is known about its impact on the SASP. The Campisi laboratory has shown that ionising radiation or oncogenic RAS-induced senescence developed a SASP regardless of expression of p16, suggesting that these are two separate pathways. However, the mechanisms behind it are not yet understood [88].

A few studies connect the SASP with reinforcement of senescence via increased ROS (Figure 3b). Acosta and colleagues have shown that inhibition of CXCR2, a promiscuous receptor that transmits signals from several CXC chemokine family members (CXCLs), including IL-8, delayed the onset of both replicative and oncogene-induced senescence and led to decreased activation of a DDR [22]. Mechanistically, the authors proposed that inhibition of CXCR2 reduced the DDR potentially by reducing ROS. β-IFN has been shown to induce senescence through ROS production and subsequent activation of the DDR, which could be inhibited with the antioxidant N-acetyl cysteine [89]. TGF-β, a family of secreted peptides that regulate a variety of processes such as proliferation, adhesion, migration, and differentiation in several cell types, has also been implicated in senescence. Inactivation of TGF-β1 secretion in mouse keratinocytes was sufficient to prevent oncogene-induced senescence [90]. In human fibroblasts, blocking TGF-β1 type II receptor (TGFBR2) activity has been shown to prevent Ultraviolet B-induced senescence and hydrogen peroxide-induced senescence [91,92]. Recently, it was demonstrated that the TGF-β induced senescence in a paracrine fashion [81]. Interestingly, neutralising antibodies or chemical inhibitors against the TGFBR2 have been shown to decrease ROS production downstream of the DDR induced in a telomere-dependent and -independent fashion [21].

Another potential link between the SASP and ROS is the fact that several studies indicate that NF-κB, the main regulator of the SASP, is also a major player in the regulation of mitochondrial function and oxidative stress (Figure 3c). Firstly, NF-κB is localised in mitochondria from yeast [93] and mammalian cells and contributes to the regulation of mitochondrial encoded genes [94]. Bakkar and colleagues reported that activation of the RelB subunit of NF-κB during myogenesis is important for mitochondrial biogenesis [95]. More recently it was demonstrated that IKKα and RelB regulate the transcription co-activator PGC-1β, a master regulator of mitochondrial function, to promote oxidative muscle metabolism [96]. Secondly, it has also been reported that NF-κB is involved in the transcriptional regulation of both nuclear-encoded anti-oxidant and pro-oxidant genes [97]. A recent study in a mouse model of type II diabetes-induced cardiac dysfunction has shown that enhanced NF-κB activity is associated with increased oxidative stress. The authors demonstrated that chemical inhibition of NF-κB alleviated oxidative stress, improved mitochondrial structural integrity, and ultimately restored cardiac function in type II diabetes [98].

In contrast, numerous reports have implicated ROS in the activation of NF-κB [99]. Both DNA binding and transactivation by NF-κB have been shown to be strongly activated by H_2_O_2_[100]. Mechanistically, evidence suggests that ROS are both cause and consequence of NF-κB pathway activation during senescence, making it challenging to establish which process occurs first. Further work is needed in order to understand the kinetics of activation of these pathways during senescence.

## Conclusions

In addition to its previously documented role as a tumour suppressive mechanism, recent evidence strongly implicates cellular senescence in ageing and age-related diseases. Both telomeric and non-telomeric DNA damage has been shown to contribute to the phenotype, with ROS playing an important role in both the induction and stabilisation of senescence. Moreover, the activation of the DDR, and the MAPK and NF-κB pathways has been shown to contribute to the regulation of both ROS and the SASP. Despite accumulating evidence suggesting that ROS and the SASP cooperate to induce and stabilise the senescent phenotype, further research is necessary to mechanistically delineate their interactions in regulating their response, and their contributions to modulating the surrounding tissue micro-environment.

## Abbreviations

DDF: DNA damage foci; DDR: DNA damage response; DSB: double strand break; IFN: interferon; IL: interleukin; NF: nuclear factor; NHEJ: non-homologous end joining; p38MAPK: p38 mitogen-activated protein kinase; ROS: reactive oxygen species; SASP: senescence-associated secretory phenotype; TGF: transforming growth factor.

## Competing interests

The authors declare that they have no competing interests.

## Authors’ contributions

CCM and JFP wrote the majority of the manuscript. GH wrote the section about telomeres and made figure schemes. All authors read and approved the final manuscript.
